# Lindy Effect in Hydrometallurgy

**DOI:** 10.1007/s40831-025-01119-x

**Published:** 2025-05-22

**Authors:** Koen Binnemans, Peter Tom Jones

**Affiliations:** 1 Department of Chemistry, KU Leuven, Celestijnenlaan 200F, Box 2404, B-3001 Heverlee, Belgium; 2 Department of Materials Engineering, KU Leuven, Kasteelpark Arenberg 44, P.O. box 2450, B-3001 Heverlee, Belgium

**Keywords:** Extractive metallurgy, Flowsheets, Hydrometallurgy, Process economics, Process engineering

## Abstract

The Lindy Effect can be formulated as: *the older the technology, the longer it is expected to last*. In this paper, we examine the historical aspects of hydrometallurgy through the lens of the Lindy Effect, aiming to understand why research efforts by academic and industrial groups seldom result in new commercial hydrometallurgical processes. We argue that many researchers, particularly in academia, fail to recognize that mining and extractive metallurgy are economic activities. Companies engaged in mining, extraction, and refining of metals must generate profits to sustain their operations. The technical feasibility of a hydrometallurgical process does not inherently guarantee its economic viability. The industrial installations in a hydrometallurgical plant are highly capital-intensive. We will demonstrate that for the development of a robust hydrometallurgical process that could become Lindy-proof in the future, it is crucial to avoid fatal flaws arising from intrinsic problems with the chemical reactions behind the process. The concept of circular hydrometallurgy and its twelve principles provides a valuable framework for assessing the robustness of new hydrometallurgical processes. A paradigm shift in hydrometallurgy is anticipated with the widespread availability of inexpensive, renewable energy. High energy costs will no longer be a prohibitive factor, allowing the development of energy-intensive processes that offer significant chemical advantages. This shift may even lead to a reconsideration of older hydrometallurgical processes that were previously deemed too energy-intensive.

**Graphical Abstract:**

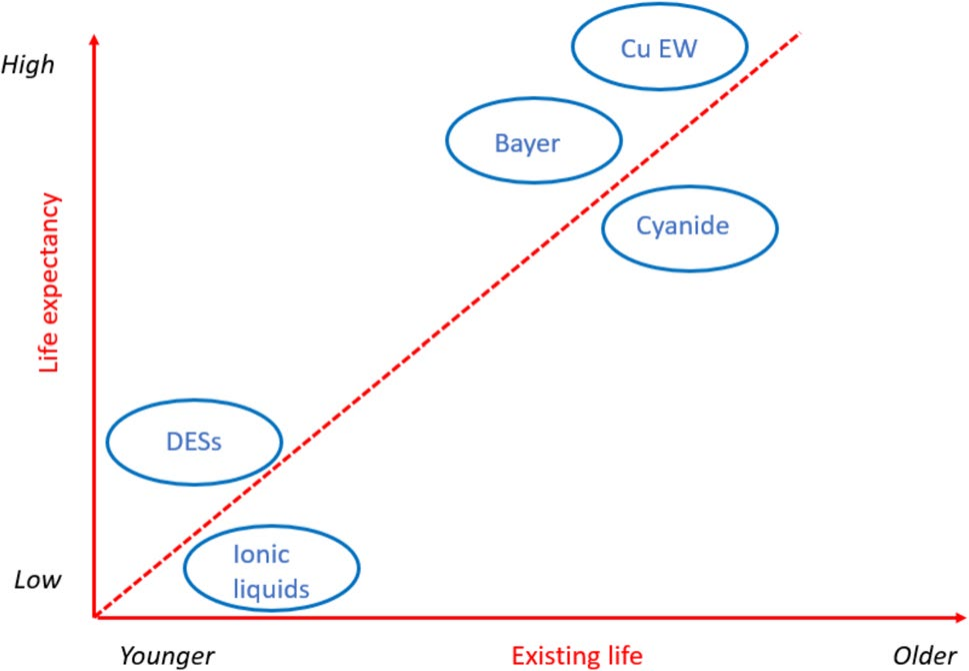

## Introduction

The past decade has seen intense research activities in the field of hydrometallurgy, largely fueled by public funding made available to mitigate the supply risk of critical raw materials (CRMs) and to meet the increasing demand for the metals required for the transition to a climate-neutral society. This research has resulted in a large number of papers in peer-reviewed journals and conference proceedings, as well as a steadily increasing number of patent applications. Most of this research has been carried out in the laboratories of universities and research institutes, and by small startup companies, but much less by larger metallurgical companies. A common theme in these papers and patents is the development of innovative hydrometallurgical processes that are touted as cleaner, more sustainable and more efficient than the best available technologies that are currently being used in industry. These new processes are often based on new reagents such as ionic liquids (ILs) and deep-eutectic solvents (DESs). However, very few, not to say none, of these highly promoted innovations have been implemented in the metallurgical industry. This is a very disappointing outcome, taking into account the enormous R&D budgets and large sums of tax payers money that has been spent to support these research activities. On the other hand, it is noteworthy that several of the well-known hydrometallurgical processes, such as the Bayer process for producing alumina from bauxite ore and the cyanidation process for gold and silver, are still in use despite being developed over a century ago (both processes were disclosed in 1887). These facts could lead to the naive assumption that the metallurgical industry is very conservative and averse to innovation. However, a closer look at the history of hydrometallurgy provides another interpretation to these observations.

The first author of the present paper has a strong interest in philosophy and cognitive sciences, and he has noticed that the practices in the field of hydrometallurgy are textbook examples of a simple heuristic (*i.e.*, a mental shortcut or a rule of thumb) that is known as the ‘*Lindy Effect*’. The ‘*Lindy Effect*’ suggests that non-perishable items have a life expectancy that increases with each day they survive. The longer something is around, the longer it is likely to continue existing. Applied to technology, the Lindy Effect can be formulated as: *The older the technology, the longer it is expected to last*.

The Lindy name derives from the famous Lindy’s Delicatessen (Lindy’s Deli) in New York. Author Albert Goldman became interested in the discussions that took place between entertainment industry veterans who frequented the restaurant and would analyze the latest televised comedy shows. They came to the conclusion that the more frequent the comedian was on the television, the shorter the comedian’s time in the spotlight…due to running out of material [1]. Goldman called this general rule ‘*Lindy's Law*’. Mathematician Benoit Mandelbrot came in his book *The Fractal Geometry of Nature* to the opposite conclusion and expressed mathematically that the expected survival of an artist’s work was, on average, lengthened as the work continued to survive [2]. He coined the term ‘*Lindy Effect*’. Mandelbrot’s concept of the Lindy effect was popularized and expanded by the Lebanese-American former option trader, mathematical statistician and bestselling author Nassim Taleb, who wrote in his book *Antifragile: Things That Gain from Disorder* (p. 318):

If a book has been in print for forty years, I can expect it to be in print for another forty years. But, and that is the main difference, if it survives another decade, then it will be expected to be in print another fifty years. This, simply, as a rule, tells you why things that have been around for a long time are not “aging” like persons, but “aging” in reverse. Every year that passes without extinction doubles the additional life expectancy. This is an indication of some robustness. The robustness of an item is proportional to its life! [3].

Aging in reverse means that its life expectancy lengthens with time. Unlike many other heuristics, the Lindy Effect (or Lindy’s Law) can be described in a formal mathematical language for use in statistics [4, 5]. Taleb coined the term *Lindy-proof* for something whose life expectancy increases with time, conditional on survival [6].

In this paper, we analyze the historical development of hydrometallurgy with the Lindy Effect in mind and try to understand why the research efforts by academic and industrial research groups hardly ever lead to new commercial hydrometallurgical processes. We will defend the thesis that many researchers, especially in academia, overlook the fact that mining and extractive metallurgy are primarily economic activities, and that the companies involved in mining, extraction and refining of metals must generate profits to survive. It is not because a hydrometallurgical process is technically feasible that it is also economically viable. For the development of robust hydrometallurgical processes that could become Lindy-proof in the future, the process must not exhibit fatal flaws due to intrinsic problems with its underlying chemistry. To determine whether the chemistry of a new hydrometallurgical process is robust, the concept of circular hydrometallurgy and its 12 principles is a useful tool [7]. A paradigm shift in hydrometallurgy might be expected when inexpensive renewable energy becomes widely available: high energy costs will no longer be a decisive factor in excluding the development of energy-intensive processes that have significant chemical advantages. We can even forecast a renaissance of older hydrometallurgical processes that are no longer in use because they were considered to be too energy-intensive.

## Lindy-Proof Hydrometallurgical Processes

**Fig. 1:**
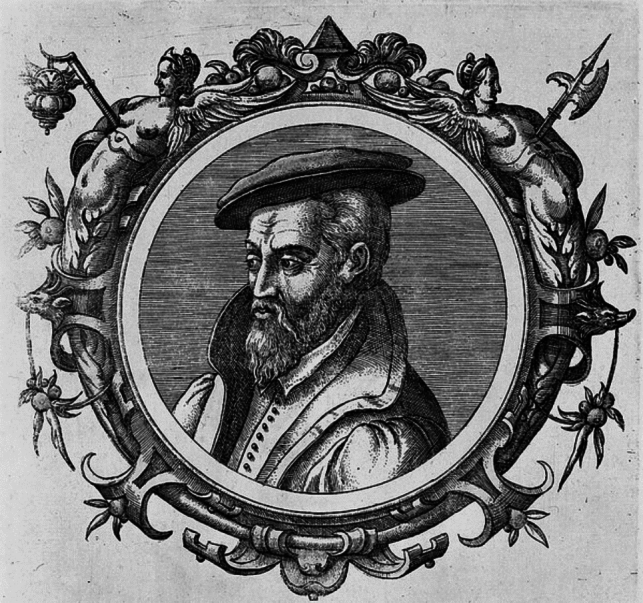
Georgius Agricola (1494–1555), author of De Re Metallica Libri XII. Source: Wikipedia

One of the most influential historical books in the field of metallurgy is undeniably Georgius Agricola’s *De Re Metallica Libri XII*, published posthumously in 1556 [8]. Georgius Agricola (1494–1555) was born as Georg Pawer (Bauer), but he Latinized his name in line with the humanistic tradition of the sixteenth century (Fig. 1). Agricola worked as physician and apothecary in the mining town of St. Joachimsthal (now Jachymov in the Czech Republic) on the Bohemian flank of the Erzgebirge/Ore Mountains, and died in Chemnitz (Germany) [9]. Agricola gathered his extensive knowledge on mining and metallurgy through a combination of practical experience and scholarly research. His job as a physician allowed him to interact closely with miners and metallurgists, observing their techniques and equipment firsthand. Agricola also visited mines and metallurgical plants regularly, documenting the processes he witnessed. Additionally, he studied classical texts such as *Naturalis Historia* of Pliny the Elder and engaged with contemporary scholars, which helped him integrate historical knowledge with his observations. The book is renowned for its detailed woodcut illustrations and thorough descriptions of mining machinery and processes. It remained the most authoritative book on mining and metallurgy for nearly two centuries.

**Fig. 2:**
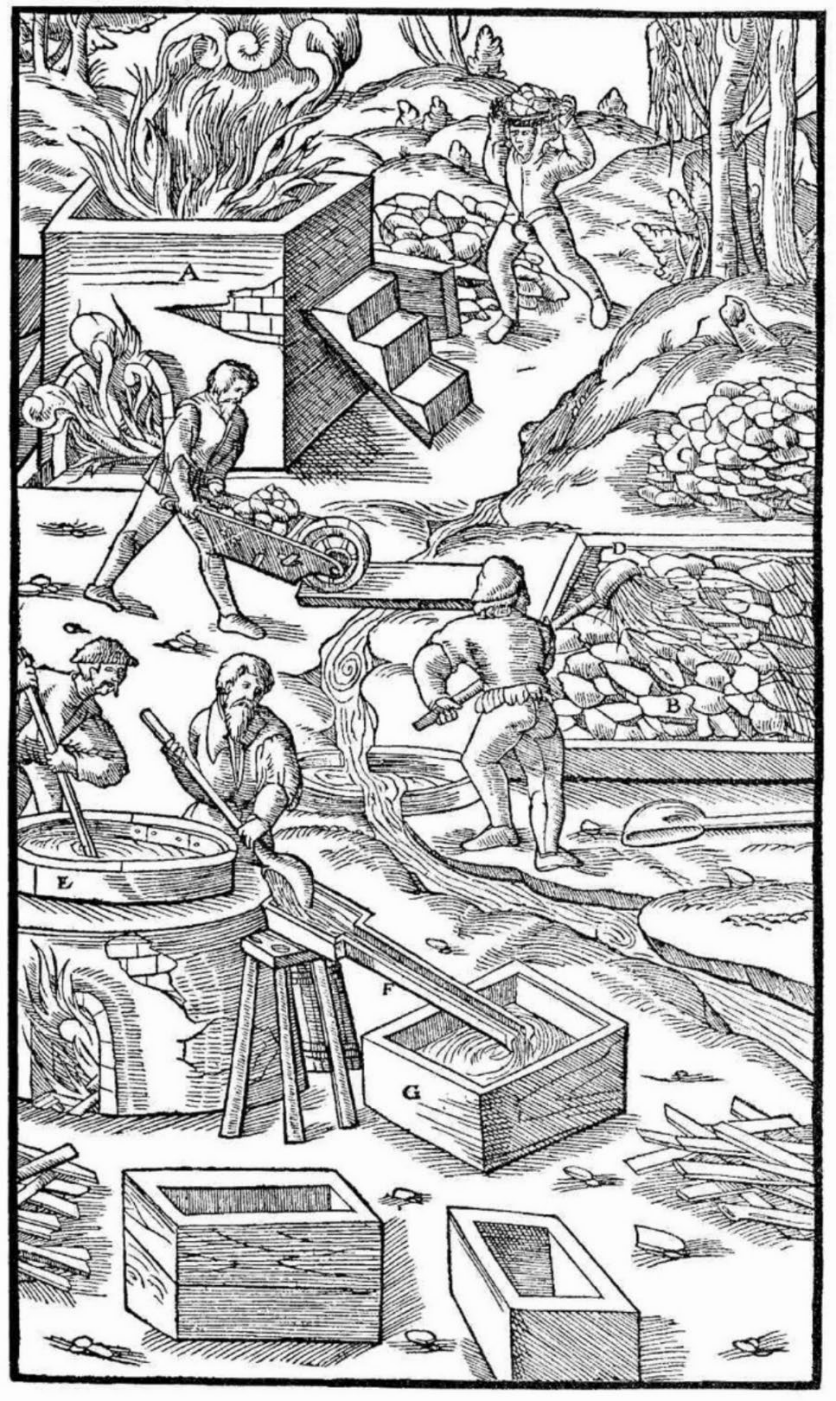
Production of alum from roasted aluminous rocks by vat leaching. **A** furnace; **B** enclosed space; **C** aluminous rock; **D** deep ladle; **E** caldron; **F** launder; **G** troughs. Reproduced from Georgius Agricola’s *De Re Metallica Libri* [8]

An English translation of *De Re Metallica Libri XII* was published in 1912 by mining engineer Herbert C. Hoover and his wife Lou Henry Hoover, a geologist and Latinist. Hoover later became the 31st US president during 1929–1932. The 1912 Hoover translation, with 638 pages with 292 woodcuts, was reprinted in 1950 by Dover Publications in New York [8]. This text and the woodcuts are accessible online for free via the *Project Gutenberg* [10]. Although this book has been published more than 450 years ago, it is still well readable, in contrast to most other books of that time. The translators have added an extensive list of footnotes with technical and historical background information and these footnotes significantly increase the relevance of the original text for modern readers. The focus of the chapters on metallurgy are obviously on pyrometallurgy, but the book is also a rich source of information on hydrometallurgy. Agricola describes the production of nitric acid and its use for separation (parting) of silver from gold. The use of *aqua regia* for the dissolution of gold is also mentioned. *De Re Metallica Libri XII* contains a detailed description of different leaching operations such as heap leaching and vat leaching (Fig. 2). Agricola describes how copper was recovered from solution by cementation of iron scrap. Agricola mentions the recovery of copper of copper-containing ores by heap leaching and that the leaching of the low-grade copper ore left exposed to the rain can be completed within 40 days. This is in fact a description of bioleaching, although it was not recognized at that time that solubilization of the copper was caused by the action of microorganisms [11]. In the book, one can read in detail how salts can be recovered from brines by evaporation, either via solar evaporation or accelerated evaporation by boiling the solutions.

### Copper Electrorefining

The *electrorefining* of metals finds its origin in the mid-nineteenth century and was a direct application of the laws of electrolysis, which have been discovered by Michael Faraday in 1833 (and published the year after) [12]. The first patent for copper electrorefining was filed by James B. Elkington in England in 1865 [13], concerning the recovery of purified copper as cathode. A second British patent granted in 1869 additionally aimed at the recovery of silver from the anode sludge [14]. A patent for this invention was granted in the USA in 1870 [15]. The rationale for this invention was to find a method for obtaining purer copper metal than the blister copper produced by pyrometallurgical processes such as fire refining, and to find an easier way to recover the silver values from the impure copper. High-purity copper metal was required for producing copper wires for the rapidly growing telegraphy industry. Cast anodes of impure copper were made by pouring the molten blister copper in cast-iron molds. A copper(II) sulfate electrolyte was used. Elkington mentions that the residue that accumulates at the bottom of the electrolysis cells, *i.e.*, the anode sludge, is rich in silver and contains some gold, tin and antimony, so that is has a considerable market value and may be sold to refiners. The texts of the first electrorefining patents are still very recognizable for modern readers, even though they are more than 150 years old. The world’s first copper electrorefinery began production in 1869 at Burry Port in South Wales, within the Pembrey Copper Works [16]. This refinery remained operational until 1912. A specimen of electrorefined copper produced by this refinery in 1880 and held by the Science Museum in London was analyzed by ICP-MS [17]. The copper sample was found to be remarkably pure: the total concentration of the 12 impurity elements analyzed was only 20 ppm. This might indicate that the copper anodes used as input for the electrorefining process were already of quite high purity. The copper electrorefining process has only undergone evolutionary changes since its introduction in 1869, with significant improvements in energy efficiency and the use of additives for better cathode morphology [18, 19].

The Moebius process, invented in 1884 for separating silver from gold, platinum and palladium by electrorefining silver anodes in a silver nitrate electrolyte, is still used today by the precious metal refining industry [20]. Electrorefining processes are so successful because they are conceptually simple and do not consume reagents (with the exception of small amounts of additives). It can be predicted that electrorefining processes are really Lindy-proof and that there are no reasons to assume that alternative processes can become serious competitors for electrorefining.

### Gold Production

**Fig. 3:**
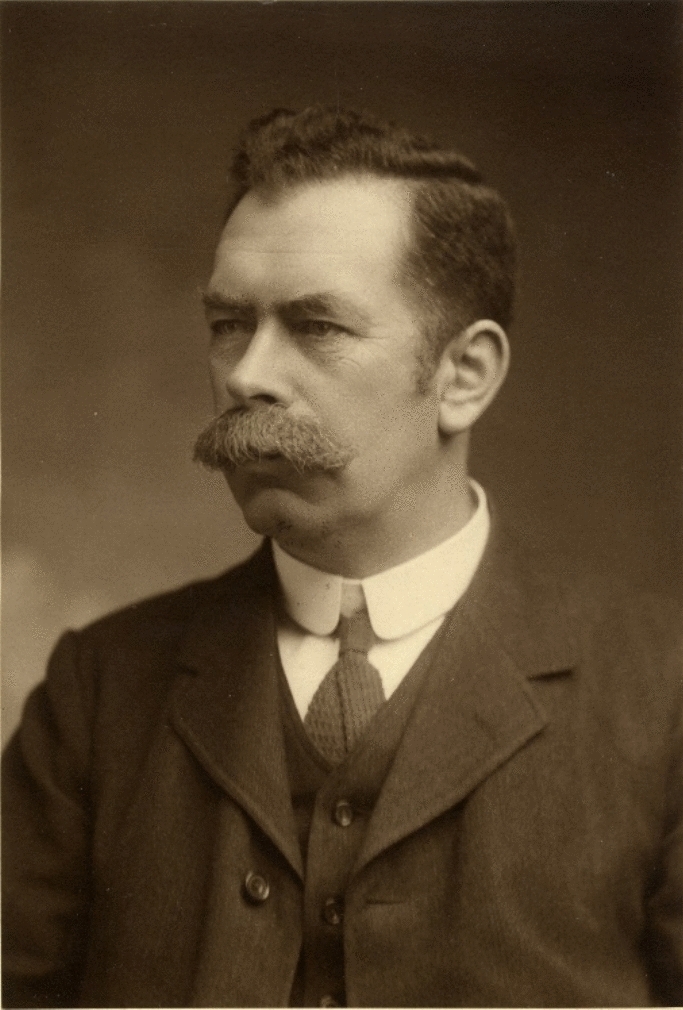
John Stewart MacArthur (1856–1920), discoverer of the cyanidation process for gold and silver. Source: Wikipedia

Swedish chemist Carl Wilhelm Scheele discovered in 1783 that metallic gold could be dissolved in a potassium cyanide solution. The solution obtained by Scheele’s method was used by Wright in 1840 in Birmingham (UK) to prepare an electrolyte bath for electroplating gold. Several chemists studied the dissolution mechanism of gold in cyanide solutions in detail and found that air was necessary to enable gold dissolution. In 1883, the Scottish chemical metallurgist John Stewart MacArthur (1856–1920) (Fig. 3), together with the two brothers Robert and William Forrest, who were his laboratory assistants, filed a patent for a process to recover gold and silver from ores. This process became known as the *cyanidation process*, in reference to the alkali cyanide reagent used [21, 22]. It is sometimes also referred to as the *MacArthur–Forrest cyanidation process*, in honor of its inventors. The cyanidation process as patented by MacArthur and the Forrest brothers involves two steps: (1) dissolution of gold from ores by an alkali cyanide solution, and (2) precipitation of gold from the pregnant leach solution by metallic zinc. The process for recovery of silver is similar to that for gold. The impact of the cyanidation process on hydrometallurgy was tremendous. The cyanidation process became an immediate success and was responsible for the doubling of gold production in the world in the two decades following its first application. For over a century, leaching gold from its ores using cyanide solution has been the dominating technology in the mining industry due to its numerous advantages: (1) it is highly specific for gold, (2) it can efficiently recover gold from low-grade ores containing as little as 1 g of gold per ton of ore, (3) dilute solutions of sodium cyanide, typically in the range of 0.01 to 0.05% cyanide, can be used, (4) the cheapest oxidizing agent, air, can be used, (5) leaching can be carried out at ambient temperatures, minimizing energy input, (6) high pulp densities of about 50% solids can be used, (7) the cyanide solutions are non-corrosive to commonly used construction materials for leaching reactors, (8) the cyanide ions can be easily oxidized to harmless oxidation products by either biological or chemical oxidation processes, (9) sodium cyanide is a relatively inexpensive reagent [23]. Despite its long list of advantages, the primary disadvantage of cyanide is its toxicity. Given the large scale at with the cyanidation process is applied in the mining industry, only very few serious accidents have occurred over the long period this reagent has been in use. However, environmental damage has resulted from mismanagement of cyanides.

Intense research efforts have sought to find safer non-cyanide lixiviants for gold. However, none of these reagents has been widely adopted by the mining industry because they are too expensive and/or have too many disadvantages [24, 25]. For instance, thiosulfate leaching didn't proceed beyond its first application at Barrick’s Goldstrike mine in Nevada, where leaching with calcium thiosulfate was used to recover gold from stockpiled complex sulfur–carbon-based ore, known as *double-refractory ore*, which is difficult to treat with conventional cyanide leaching. Whereas the leaching process was well developed, the thiosulfate technology was immature with respect to the process steps after leaching, the fate of some species in solution, water purification requirements, reagent availability, microbial impacts on the reagents, recyclability of reagents, degradation through sunlight, etc.

### Alumina Production

**Fig. 4:**
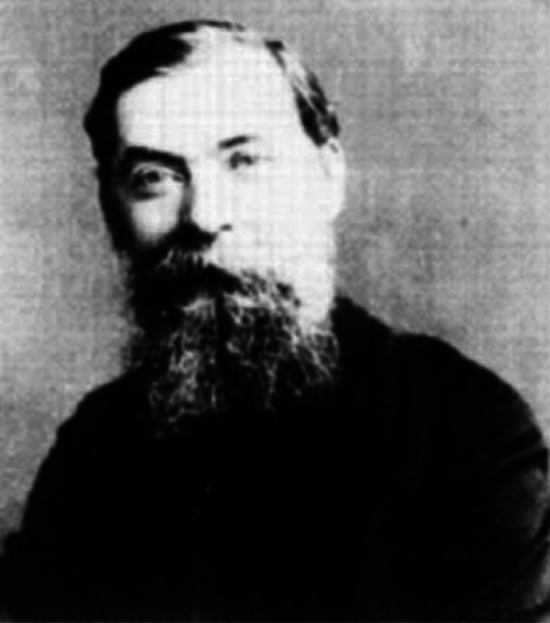
Karl Josef Bayer (1847–1904), inventor of the Bayer process for the production of alumina from bauxite. Reproduced with permission from reference [26]. Copyright Elsevier 2005

**Fig. 5:**
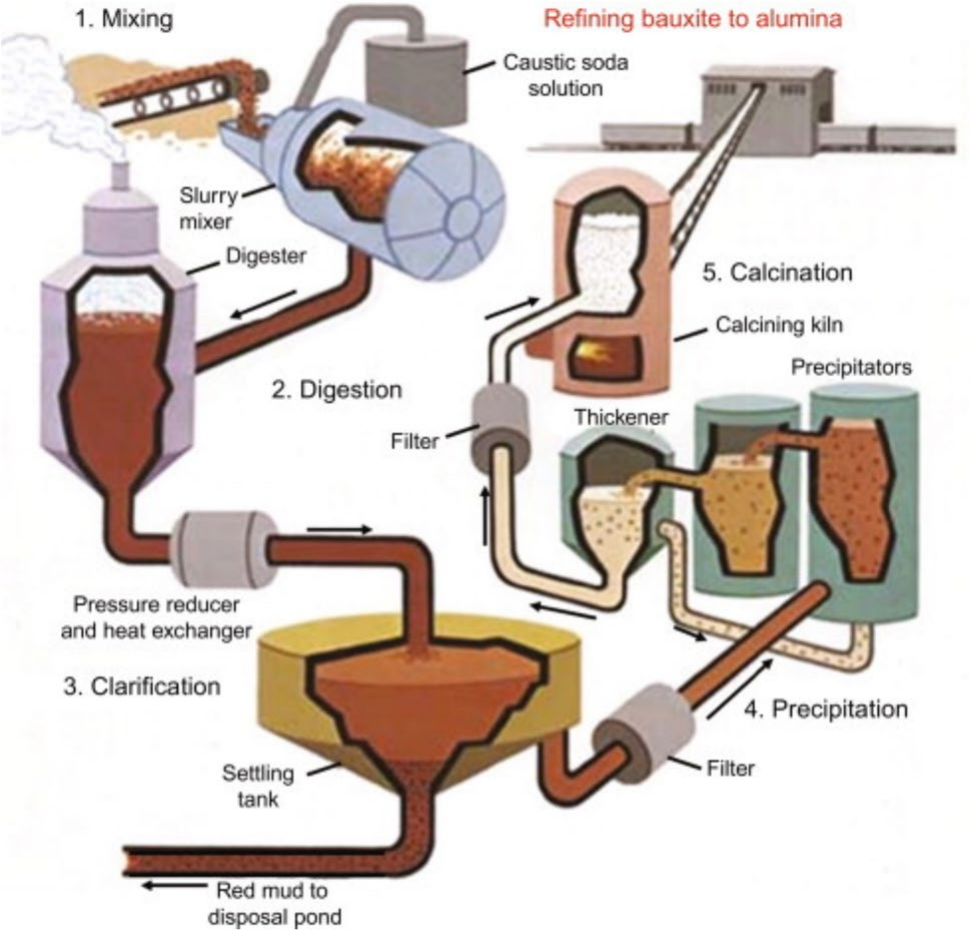
Schematic presentation of the Bayer process for the production of alumina from bauxite. Reproduced with permission from Reference [29]. Copyright Elsevier 2014

The *Bayer process* for producing alumina from bauxite ore was developed between 1888 and 1892 by the Austrian-born chemist Karl Josef Bayer (1847–1904), who was working in Russia at the time (Fig. 4) [26]. The process involves two steps: (1) the pressure leaching of bauxite with sodium hydroxide solution to obtain sodium aluminate solution, with disposal of the leaching residue (bauxite residue or red mud), and (2) the precipitation of pure alumina from this solution by seeding with fine crystals of Al(OH)_3_ (Fig. 5) [27–29]. The Bayer process, involving both pressure leaching and precipitation by seeding, is used today in practically the same way as it was invented by Bayer more than 100 years ago. However, significant improvements in the engineering aspects of the process have been made to reduce costs. These improvements include much more efficient heat recovery, larger autoclaves and precipitation tanks, continuous operation, and increased automation. The Bayer process has several appealing features that have contributed to its long-term success in the alumina industry. First of all, the aluminum minerals can be selectively leaching from the bauxite ore, leaving the non-aluminum minerals in the residue (the bauxite residue). Secondly, the Bayer process can consistently produce highly pure alumina. The technical specifications for the quality of the alumina feed for the Hall-Héroult process for aluminum metal production are very strict; the maximum allowed concentrations of impurities (Ca, Mg, Si, Fe…) in the alumina are very low to minimize the accumulation of impurities in the electrolyte bath of the electrolysis cells, allowing them to remain in use for a sufficiently low time (5 to 8 years). Thirdly, the sodium hydroxide reagent used to dissolve the aluminum minerals in the form of sodium aluminate is regenerated when alumina crystallizes out of the sodium aluminate solution, significantly reducing reagent consumption.

The generation of substantial quantities of bauxite residue (red mud) during the Bayer process continues to be a significant concern. Despite extensive research efforts aimed at extracting valuable base and minor metals from the residue and finding applications for the inorganic matrix, economically viable solutions have yet to be developed [30, 31].

### Zinc Recovery

Cooper et al*.* evaluated 11 different potential flowsheets against a total of 22 different criteria to define the optimum zinc refinery flow sheet in an engineering design process [32]. Criteria included CAPEX, OPEX, energy consumption, water consumption, carbon footprint, health and safety, technical risk, zinc recovery yield, and feed grade flexibility. The winner was the traditional *Roast–Leach–Electrowinning* (RLE) process, with all other (more) novel technologies lagging far behind due to flaws such as high CAPEX, high OPEX, high energy consumption and low zinc recovery. “Unproven at commercial scale” was a recurrent negative comment for several of the alternative flowsheets. Interestingly, the traditional pyrometallurgical technology for processing zinc sulfide concentrates, the *Imperial Smelting Process* (ISP), was ranked ninth [32]. Obviously, the ranking of the flowsheets strongly depends on the scores and their weighing factors that assigned to each of the different criteria. The RLE process was first successfully demonstrated in a 10-ton zinc per day pilot plant by the Anaconda Copper Mining Company in Anaconda, Montana (USA) in 1915 [33, 34].

### Cheap and Simple Reagents

When analyzing the traditional processes that have been described above, it becomes evident that all these processes use cheap, simple chemical reagents, and the basic chemistry behind the processes has not changed over time. The processes have been strongly improved since their invention, but these changes are largely related to engineering aspects, such as making the processes more energy-efficient through the use of heat exchangers, or increasing automation, including the extensive use of sensors and other analytical tools to monitor the industrial process.

### Solutions to Non-existent Problems

One important condition that must be fulfilled to convert a successful R&D hydrometallurgical project into a profitable business is that the proposed process must provide a solution to a real problem [35]. This may seem so obvious that many readers might consider it not worth mentioning. However, reality often tells a different story. Researchers in academia often try to find solutions for problems that don’t really exist in the real (industrial) world. Much of the academic research is not relevant from an industrial perspective. One reason for this mismatch is poor communication or exchange of ideas between academics and industry. This is partly because few people have worked in both academia and industry environments within the field of hydrometallurgical research or have close collaborations with industry. Consequently, challenges include understanding the drivers, priorities, limitations, timelines and even the language of the two groups. Another problem is that researchers in academia are often too focused on novelty and discovery for its own sake, with the main objective being to publish papers in conference proceedings of peer-reviewed journals rather than solving real industrial problems. The claims made by academic researchers—often made to attract research funding—are too broad and sweeping. They frequently capture media attention but tend to die a silent death when it is realized it may be an extremely niche application only relevant to a very specific ore.

### Replacing Sulfuric Acid

One striking example of solutions to non-existent problems is the search for “greener” alternatives for sulfuric acid in hydrometallurgy. Several authors have expressed concerns about the environmental impact of sulfuric acid as a lixiviant and propose replacing it with organic acids [36, 37]. Naturally occurring acids, such as citric acid, are promoted as more ecological alternatives for leaching metals from low-grade ores, industrial process residues or other metal-containing solids. For instance, citric acid has been investigated for the recovery of nickel from low-grade laterite ores [38], lead and copper from metallurgical slags [39], and metals from waste printed circuit boards [40]. It has also been proposed for recovering cobalt, nickel, manganese, and lithium from spent lithium-ion batteries [41]. Citric acid seems to be an ideal reagent for hydrometallurgy, because it is relatively cheap, non-toxic, biodegradable, and a renewable chemical that can be produced in large quantities by microbial fermentation of carbohydrate substrates such as beet and cane molasses by fungi such as *Aspergillus niger* [42, 43]. However, a closer examination of the production process of citric acid reveals that claims of its superior sustainability compared to sulfuric acid are difficult to defend. In the most commonly used commercial process for recovering citric acid from the fermentation broth and subsequent purification, the liquor containing citric acid is first filtered to remove mycelia and other suspended solids. The filtrate is then heated with a calcium hydroxide slurry (slaked lime) to precipitate hydrated tricalcium citrate, Ca_3_(C_6_H_5_O_7_)_2_·4H_2_O. The precipitated calcium citrate is filtered, washed with water several times, and treated with sulfuric acid to liberate the citric acid. The CaSO_4_·2H_2_O (gypsum) formed is separated from the citric acid in solution by filtration. The citric acid can be further purified using an activated carbon column, and finally, citric acid crystals are obtained from the solution in a vacuum crystallizer [44]. The production of citric acid consumes stoichiometric amounts of lime and sulfuric acid, requires large amounts of water and generates about 2.5 tons of waste per ton of citric acid. The energy consumption in the process is also substantial. Although solvent extraction has been proposed as a possible alternative to the classical method, it has its own issues [45]. Given the large amounts of sulfuric acid consumed to prepare the citric acid intended to replace sulfuric acid for leaching in hydrometallurgy, it is clear that such a replacement does not make sense from a sustainability perspective. Sulfuric acid has many attractive properties that make it the acid of choice in many hydrometallurgical processes. First of all, it is very cheap compared to other acids. It is compatible with electrorefining and electrowinning processes, and for electrowinning it has the advantage that oxygen gas is formed at the anode in sulfate electrolytes rather than chlorine gas in chloride electrolytes. Unless in concentrated form, sulfuric acid is only moderately corrosive to commonly used reactor construction materials such as stainless steel. Sulfuric acid is a byproduct of processing sulfidic concentrates through smelting or roasting and can be produced on-site. The production of sulfuric acid is highly exothermic, and the heat generated can be utilized to produce steam for heating other processes or to drive a turbine for electricity generation. Finally, it should not be forgotten that sulfuric acid is formed during bioleaching with autotrophic bacteria. Only in cases where sulfuric acid does not work well, such as in lead hydrometallurgy due to the low solubility of PbSO_4_, must alternatives such as hydrochloric acid or methanesulfonic acid (MSA) be considered [46].

### Ionic Liquids and Deep-Eutectic Solvents

The literature on the application of ionic liquids (ILs) and deep-eutectic solvents (DESs) in extractive metallurgy is full of examples addressing non-existent and imaginary problems. Simply reading the introductions of randomly selected papers on this topic quickly reveals a general tendency among authors to demonize current practices in hydrometallurgy and to present these organic solvents as a panacea. However, in a recent opinion paper, we provided eight reasons why it is very unlikely that these neoteric solvents will ever be adopted in an industrial metallurgical flowsheet: (1) issues with high viscosity; (2) limited chemical stability under the conditions of metallurgical processes; (3) difficulties with recycling and reuse; (4) a lack of demonstrated unit processes and flowsheets on the pilot scale; (5) insufficient material−property data available for engineering purposes; (6) the administrative burden of obtaining licenses and safety permits; (7) very high costs for large-scale operations; (8) minimal added value compared to state-of-the-art hydrometallurgical processes [47].

### Recycling (Market) Issues

From an economic point of view, it makes little sense to develop hydrometallurgical flowsheets for metal-containing streams that are available only in very small volumes or that will be no longer available in the near future. This is particularly the case for certain types of waste streams for which dedicated flowsheets have been developed. Many examples can be found in the literature on recycling of rare earths (or rare-earth elements, REEs). In our 2013 review on rare-earth recycling, we stated that it does not make sense to develop recycling schemes for the recovery of rare earths from laser crystals, because the volumes are so small [48]. However, this statement can now be extended to many other REE-containing waste streams or end-of-life products, such spent glass polishing powders or luminescent powders in cathode-ray tube (CRT) screens. The recovery of rare earths from lamp phosphor waste was a hot research topic a few years ago because this waste fraction contains large amounts of europium, terbium and yttrium, which were considered very critical metals at that time. However, fluorescent lamps rapidly disappeared from the market because they were replaced by light-emitting diode (LED) lamps. This means that the amounts of lamp phosphor waste will steadily decrease and less and less will become available in the future. When rare earth prices were high, particularly in the period 2011–2015, there was significant interest in developing hydrometallurgical processes for recovery of rare earths from fluorescent lamp phosphor waste [49]. Despite substantial public funding and intense research efforts, none of these processes have led to new industrial processes that have survived until today. Although the market potential for new metallurgical processes to recover rare earths from lamp phosphors is virtually zero, this remains a very active research field [50, 51]. Spent nickel metal hydride (NiMH) batteries is another example of a waste stream for which dedicated hydrometallurgical flowsheets have been developed [52], but none have been commercialized due to poor process economics and the unavailability of sufficient input material to be processed.

### Metal Recovery from Low-Grade Industrial Process Residues

While the hydrometallurgical processes described in the previous paragraph were doomed to fail commercially because that were developed for waste streams available only in small volumes or that have disappeared from the market, there are also waste materials and industrial process residues produced in enormous volumes and for which large historic stocks exist. Examples include phosphogypsum from the phosphate industry, iron-rich sludges from the zinc industry (jarosite and goethite), and the bauxite residue (red mud) produced in the Bayer process of alumina production from bauxite ore [53–56]. These materials contain concentrations of critical raw materials (CRMs) such are rare-earth elements, indium and germanium, which attract interest from researchers and investors when the prices of these CRMs are very high and/or China announces new export quotas. The process economics of these processes are often assessed based on peak prices that are an order of magnitude higher than the 5-year average prices. It goes without saying that all these business plans can be discarded when prices decrease to normal levels. Moreover, it is often overlooked that extraction of the valuable trace metals from these industrial process residues does not solve the problems associated with storing the huge volumes of these waste materials in tailing ponds or dry stacks. A good example is the research on the recovery of scandium from bauxite residue. Depending on the source of the bauxite, the resulting bauxite residue contains between 60 and 120 g/ton of scandium, or on average 100 g/ton [57]. This means that when 100 g of scandium is extracted from 1000 kg of bauxite residue, most of the original mass of the bauxite residue remains in the leaching residue. This practice clearly does not solve the storage problem and the accompanying environmental issues of the bauxite residue. Even worse, there is the risk that toxic trace elements become even more mobile in the leach residue of scandium recovery than in the original bauxite residue. It only makes sense to extract scandium from bauxite residue if solutions are also found for the major components present in the bauxite residue, primarily the iron. This is the (near-)zero waste valorization philosophy [53]. While this looks good on paper, often the processing costs for extracting the different metals from the bauxite residue are higher than the revenues generated by selling these metals or materials. Moreover, there is often no local market that can absorb these materials. For instance, this is the case for cementitious binders made from bauxite residue.

### Hydrometallurgical Copper Smelter

The failure of the concept of the *hydrometallurgical copper smelter* for the treatment of high-grade sulfide concentrates examplifies how hydrometallurgical processes did not become commercially successful because the problem they aimed to address was solved by a superior competing technology, being modern copper smelters [58, 59]. For centuries, pyrometallurgical plants processing sulfidic ore concentrates had a very negative environmental impact because the SO_2_ formed by oxidation of the sulfides was vented into the atmosphere, causing severe pollution in the surrounding region. A well-known effect was acid rain, which had detrimental effects on forests. The growing environmental awareness in the 1960s led to Clean Air Acts in many countries to reduce air pollution. This legislation incentivized many research programs in universities, research institutes and companies to develop hydrometallurgical processes as alternatives to the smelting of copper sulfides, particularly chalcopyrite. Most of these processes involved oxidative leaching of the ores and concentrates, mainly in acidic media (H_2_SO_4_, HCl or HNO_3_), with a strong focus on chloride hydrometallurgy, although oxidative ammonia leaching was also investigated. Every imaginable oxidizing agent was tested. The main questions were what to do with the sulfur and the iron. Sulfur can be oxidized to sulfate, which must be removed from the circuit by precipitation of gypsum with lime, resulting in a lot of contaminated gypsum waste that needs to be landfilled. A better option is to oxidize the sulfur to elemental sulfur that can be recovered from the PLS, for instance by froth flotation. However, there is not always a sufficiently large market for this sulfur. An even larger problem is what to do with the iron in chalcopyrite (CuFeS_2_). For every mole of copper in chalcopyrite, there is one mole of iron. In *pyrometallurgical processes*, the iron reports to the slag phase as fayalite, iron(II) silicate (Fe_2_SiO_4_). Fayalite slag is a stable, inert material that can be safely disposed of or used in building materials. On the other hand, iron is precipitated from hydrometallurgical solutions in the form of iron-rich sludges such as jarosite or goethite. These are much more reactive than fayalite slag and must be stored in tailing ponds, which need to be carefully monitored to prevent environmental pollution. If iron is already a major issue in the zinc metallurgy were the maximum Fe/Zn ratio is about 1/5, it is understandable that iron is even much more problematic in copper metallurgy where the Fe/Cu ratio is 1/1 [60]. Although the research activities resulted in hundreds of scientific papers and patents, most of this body of research on copper sulfide leaching did not proceed beyond the stage of laboratory bench studies. A few of the most promising processes reached the pilot plant stage, but only two made it to full-scale production plants. These were the Arbiter process and the CLEAR process. These started up at industrial scale in 1974 and 1978, respectively. The *Arbiter process*, named after Nathaniel Arbiter, the technical director of the Anaconda company that developed the process, was based on ammonia chemistry and involved leaching chalcopyrite with an ammoniacal solution using pure oxygen as oxidizing agent [61]. Copper was recovered from the pregnant leach solution by solvent extraction and electrowinning. Duval’s chloride-based *CLEAR process* involved the leaching of chalcopyrite concentrates in iron(III) chloride solutions and electrowinning copper as a powder in a one-electron process from copper(I) chloride solutions. CLEAR stands for *Copper Leach, Electrowinning and Regeneration*. Although commercial plants were established for both the Arbiter and CLEAR process, neither proved sustainable and both were shut down after only a few years [62]. They could not compete economically with copper smelters, which had modernized to enhance their environmental performance, and had developed new smelting technologies such suspension smelting (flash smelting) and bath smelting (e.g. ISASMELT technology) [19]. These new technologies are much more efficient than the older technologies and the SO_2_ gas is recovered and converted into sulfuric acid. Despite all this extensive research and development, pyrometallurgical processing of copper sulfide concentrates continues to dominate the copper industry today, and it is unlikely that hydrometallurgical processes will be able to compete with these superior technologies for the processing of rich and clean copper sulfide concentrates. However, there might be a role for hydrometallurgy in the future to process “dirty” concentrates, such as those rich in arsenic which are difficult to treat in a smelter, and complex polymetallic ores from which it is difficult to make concentrates by minerals processing [63]. Also for small-scale operations a hydrometallurgical process could be suitable, because it reduces the CAPEX and the costs of transportation of copper concentrates to a remote smelter.

We saw a second wave of research toward the development of a hydrometallurgical copper smelter in the 1990s and 2000s. An example is the chloride-based HYDROCOPPER™ process of Metso (formerly Outotec) for copper concentrates [64, 65]. A demonstration plant was operated in Pori Finland in 2008 to process 200 tons of copper concentrate of the Zangezur copper mine of Kajaran (Republic of Armenia), and pre-engineering for a HYDROCOPPER plant for the Mongolian Erdenet Mining Corporation was carried out, but no further information on commercialization of this process is available in the open literature after 2008 [66].

A more recent development is the Albion Process™, which is being developed by Glencore since 1994 [67]. This process treats sulfidic concentrates by a combination of ultrafine grinding and oxidative leaching at atmospheric pressure. The first commercialization of the process happened at Sable in Zambia for treating copper/cobalt concentrates [68].

## Apparent Conservatism in the Metallurgical Industry

The development of a commercially successful metallurgical process generally proceeds in a similar pattern [35]. First, the problem is analyzed and a general approach is formulated. The process idea undergoes a preliminary economic evaluation to ensure that, if everything works ideally, it represents a significant improvement over known alternatives or existing practices. Secondly, after passing these initial tests, laboratory experiments are conducted to verify the soundness of the chemistry, determine deviations from the ideal behavior, and generate basic engineering data for specifying pilot equipment. Critical data include: (1) reaction kinetics as a function of controllable variables (temperature, reagent concentration, agitation level, particle size distribution…), (2) behavior of the process products, particularly regarding phase separations, and (3) impact of residual materials (reagents and impurities) on subsequent steps or products. Thirdly, if the process proves successful in the lab, a feasibility study is conducted, typically involving pilot plant work to finalize the flowsheet, establish operating criteria, and address any equipment design issues, followed by sufficient engineering to provide accurate CAPEX and OPEX estimates. Bench work may continue during this phase to support process development. Fourthly, once feasibility has been established and management approval is obtained, detailed engineering and construction are carried out. Finally, the new plant goes through startup.

For researchers in academia, it might sound surprising to carry out a simple economic evaluation of the process prior to laboratory experiments. In academia, the primary focus is often on scientific discovery and innovation, which can sometimes lead to overlooking practical economic considerations. However, in the industrial context, the feasibility of a project is heavily influenced by its potential profitability. Conducting a preliminary economic evaluation before diving into laboratory experiments is crucial because it helps determine whether the process can be financially viable under ideal conditions. If initial cost calculations indicate that the process would not be profitable, even in the best-case scenario, it is impractical to invest further time and resources into its development. This approach ensures that only economically promising projects proceed to the experimental stage, thereby optimizing resource allocation and increasing the likelihood of successful commercialization. In essence, integrating economic assessments early in the research process aligns scientific endeavors with market realities, fostering innovations that are not only technically sound but also economically sustainable [69].

Hydrometallurgy, like any industrial process, is fundamentally an economic activity. Metallurgical companies, like all other companies, must prioritize profitability to ensure their survival and growth. This means that while technological advancements and environmental considerations are important, the ultimate goal is to develop a cost-effective and efficient process that can compete in the market. A responsible/sustainable process that is not competitive makes no sense in the real world. Profitability allows companies to reinvest in research and development, adopt new technologies, and expand their operations. Without a focus on economic viability, even the most innovative metallurgical companies would struggle to be survive in the long term. Therefore, balancing technical efficiency with economic performance, while also including environmental sustainability, is crucial for the success of metallurgical enterprises.

The metallurgical industry is known to be conservative and reluctant to embrace new technologies. This cautious approach is related the high costs and risks associated with implementing unproven technologies in large-scale operations. As discussed above, the metallurgical industry often relies on established processes that have been refined over decades, ensuring reliability and efficiency. The significant investments that made in the existing infrastructure also make it challenging to justify the transition to newer technologies. The cost of building a new hydrometallurgical plant can be very high, in the order of hundreds of millions of USD. Failures in the design stage can be very expensive and can even lead to bankruptcy of smaller companies. Therefore, to minimize financial risks, no new hydrometallurgies plants will be built based on new technologies unless there are several examples of successful existing plants applying the same or similar technology. The existence of one successful operating plant is often not sufficient to convince investors. Investors want to see several successful examples. In terms of *Technological Readiness Levels* (TRLs), TRL 8 is not sufficient; what is required is TRL 9 [70]. Even for innovations that involve only adjustments of running hydrometallurgical processes, industrial companies want to see successful implementation in facilities similar to their own, before committing to expensive and time-consuming retrofits. As a result, innovations in metallurgy tend to progress slowly, with new advancements being adopted only after extensive testing and validation.

Due to the financial risks linked to constructing and operating hydrometallurgical plants based on unproven breakthrough innovations, companies often prioritize incremental innovations and continuous improvements. The goal is to reduce operating costs, which can be achieved through process enhancements that lower reagent consumption, increase energy efficiency, and implement more automation, thereby reducing the need for personnel.

Since there are not many chemists or metallurgists in the corporation's upper levels of senior executives and managers, the chemical understanding and appreciation of executive leadership teams is limited, so that there is very little willingness to take on chemistry-related risks. The lack of understanding of chemistry among company executive leaderships limits apatite to changes in process chemistry. For brownfields projects, the view is normally of trying to retrofit new technologies and making marginal changes. This is often inherently difficult as one is force-fitting a new chemistry to a metallurgical plant designed for another purpose. There are very few greenfield plants built. When they are sponsored by a stock exchange listed company, there is very limited appetite for new technology among technology (and chemistry)-ignorant shareholders.

### Copper L-SX-EW

The copper *Leach−Solvent extraction–Electrowinning* (L–SX–EW) process is a good example of a process where an entrepreneur dared to test a technology on an industrial scale that was still unproven at commercial scale at that time [58] [71]. In the 1960s, the wealthy young American Maxie Anderson owned a small copper mine, the Bluebird Ranchers Mine, in Miami, Arizona. Without shareholders and a board of directors to worry about, Anderson was willing to invest his own money to fund the very risky step of introducing a radically different technology into an industry where the basic methods of making copper had not changed significantly since the Bronze Age. The first commercial copper SX–EW plant was installed at the Bluebird mine in 1968 to treat a PLS obtained by heap leaching of a low-grade oxidic copper ore. The LIX64 extractant was developed by General Mills, with Joe House as one of the main driving forces behind General Mills’ solvent extractant R&D program, and test work was done by Hazen Research Inc. [72]. Previously, copper was recovered from the PLS containing about 1 g/L Cu by cementation with iron scrap. This plant was quite small, not much more than a large pilot plant, producing about 6000 tons of cathode copper per year, less than a month’s production from a modern, medium-sized copper smelter. However, its influence on the copper industry cannot be underestimated. After commercialization of the L–SX–EW process at Bluebird, several new L–SX–EW operations were built in quick succession, notably at Bagdad Mining Company in Arizona, not far from the Bluebird mine in 1970, at Nchanga Consolidated Copper Mines Tailings Leach Plant (TLP) in Chingola, Zambia, in 1973, and at Anaconda’s Twin Buttes in Arizona in 1975. Steady adoption continued through the 1970s and began to accelerate in the 1980s at the Lo Aguirre plant in Chile and at several Phelps Dodge properties in the USA. In the 1990s, implementation of the process mainly took place in Chile, where the production increased more than tenfold from 1991 to 2001. Copper solvent extraction technology has evolved and adapted to currently produce about 4.0 millions of tons of cathode copper per year, corresponding to approximately 18% of global primary production [71].

Maxie Anderson’s contribution to the development of hydrometallurgy cannot be underestimated, even though he did not develop the L–SX–EW process himself. With substantial personal capital, no shareholders, and a willingness to take financial risks, he decided to build the first commercial solvent extraction plant for copper. This plant demonstrated the technological and economic viability of the new hydrometallurgical technology. Joe House recounted the initial negative responses to the concept of using solvent extraction for copper, noting that no one in the major copper mining companies believed in it. House mentioned that one director of research predicted at a metallurgists’ meeting that solvent extraction would never recover a pound of copper, a statement that was met with applause [72].

### Nickel Metallurgy

In nickel metallurgy, the Moa Bay plant in Cuba demonstrated that the High-Pressure Acid Leaching (HPAL) process for processing nickel laterites can be successfully operated on a large industrial scale [73, 74]. This plant has been operational since 1959, and over time, many improvements have been made to address weaknesses in the process [75, 76]. This has given other major investors enough confidence in the technology to construct similar plants elsewhere, although starting up an HPAL plant is still not straightforward. It often takes years for an HPAL plant becomes operational at full scale, especially if on-site refining of nickel and cobalt is carried out, for instance by solvent extraction, rather than simply producing Mixed Sulfide Precipitate (MSP) or Mixed Hydroxide Precipitate (MHP) to be sold to other refineries.

### Chicken-and-Egg Dilemma

Hydrometallurgy faces a classic *chicken-and-egg* dilemma: many promising hydrometallurgical processes fail to advance to industrialization because they are considered unproven at commercial scale. Yet, without someone willing to take the risk and demonstrate these processes on a large scale, they will remain unproven. The metallurgical industry needs more pioneers like Maxie Anderson, who are willing to invest and take risks, or who have skin in the game. The development of hydrometallurgical processes is particularly risky because many issues, such as impurity buildup in aqueous streams, stability issues with extractants in solvent extraction, scale formation, and equipment corrosion, often do not become apparent during pilot plant tests or the early stages of plant operation. These challenges can significantly impact the viability and success of scaling up new hydrometallurgical technologies, making it crucial for the industry to support and encourage risk-takers who can push these innovations forward.

Successful hydrometallurgical processes are widely reported, but much less is written about failed projects [77]. This is unfortunate because one can learn a lot by studying and thoroughly understanding their failures. Knowledge of failed projects in the history of hydrometallurgy could prevent the pursuit of bad research ideas or making similar mistakes. A complete analysis of the causes of failure is often not done, the specific causes and sequences are not well understood and, worse, the details are poorly known within the developers’ staffs and only rarely are they communicated to others. Therefore, Southwick recommends to follow the “fatal flaw” route, which takes a concerted, objective and thorough effort and a deep understanding of how processes work, or don’t work, and how to conduct and solve equipment and design scale-up issues [77]. A critical and objective analysis is made of the technology itself, looking carefully at past efforts with similar processes and equipment (perhaps from other industries even), examining pilot and other tests for a thorough understanding of just how the process can succeed, which operating problems arise, what causes them and how to resolve them. It is important to note that virtually all of the problems that lead to technology failure are often already seen in the earlier small-scale laboratory tests and experiments, but not recognized and not taken into account. The focus in the literature on successful hydrometallurgical processes is an illustration of the so-called *survivorship bias*, which is the logical error of concentrating on entities that passed a selection process while overlooking those that did not [78]. This can lead to overly optimistic beliefs about the success rate of hydrometallurgical processes because multiple failures are overlooked. Reasons for failures of projects include: (1) poor understanding of the market requirements, (2) false confidence in the technology, (3) inadequate testing of the raw material, and application of processing conditions that were inherently unsafe, (4) improper assessment of the inherent advantages of past developments, (5) incomplete and superficial assessment of prior pilot plant and demonstration plant tests, (6) an assumption that the commercialization model of “build it and we will make it run” was viable, and (7) using a technology whose technical fatal flaws had already been identified [77]. In many of the failed projects, continuous testing at the pilot or demonstration scale was not carried out, and very optimistic CAPEX and OPEX were generated on the basis of very limited process engineering design criteria [79, 80].

## When the Chemistry Is Not Right

As discussed in the previous section, numerous factors contribute to the failure of hydrometallurgical R&D projects in achieving economic viability. These failures can be due to intrinsic factors, such as issues with the chemistry of the process or processes that are excessively energy-intensive, as well as external factors, like low metal prices in the market, high energy or reagent costs, or unstable political environments. When a process fails due to chemical issues, it becomes particularly challenging to transform the flawed process into a profitable one. Some reagents are so expensive that it is unlikely that they will ever be used in industrial processes. A good example are the ionic liquids and deep-eutectic solvents [47]. Serious issues with extensive scale formation in pipes and on reactor walls can make processes uneconomical. The same can be said for corrosion problems, as often encountered in chloride-based processes.

A common problem encountered is the difficulty in achieving efficient solid–liquid separation. Slow solid–liquid separation is a significant challenge in hydrometallurgy, not only for separating the solid residue from the pregnant leach solution after leaching, but also for the removal of precipitates formed during solution purification. The difficulties arise from poor settling characters of fine solid particles, the formation of gelatinous precipitation, or clogging of the filter media.

Developers of hydrometallurgical processes often face serious issues with solvent extraction processes. For instance, difficult to strip impurities can gradually build up in the solvent, leading to a gradual decrease of the solvent capacity. A well-known example is the poisoning of hydroxyoxime extractants by cobalt(III) in processes for solvent extraction of copper [81]. Another example is the poisoning of bis(2-ethylhexyl)phosphoric acid (D2EHPA) by iron(III) [82]. Solvent losses can occur due to the solubility of the extractant or diluent in the aqueous phase, or through the entrainment of organic phase droplets in the aqueous phase. While entrainment can be minimized with proper design of the solvent extraction circuit and skilled operation of the plant, controlling solubility losses are more challenging. This often requires treatments such as diluent washing of the raffinate or the use of an activated carbon column, incurring extra costs. Although most commercially used extractants initially appear to have low solubility in water, significant extractant losses can accumulate over time.

Several extractants, such as Cyanex 301 and 302, have been withdrawn from the market due to serious stability issues, although these extractants seemed to have a bright future at the time of their introduction [83]. Degradation products from these extractants can negatively impact phase disengagement times. An example of long-term operational issues includes the cobalt-catalyzed oxidation of the diluent in cobalt/nickel solvent extraction circuits using Cyanex 272 extractant. This oxidation can adversely affect the cobalt/nickel separation factors, with detrimental effects becoming apparent only after extended periods of operation [84]. Feed solutions with a high concentration of suspended solid particles might cause issues with crud formation in solvent extraction circuits. Crud formation does not only lead to major solvent losses, but also to downtime of the solvent extraction plant when the circuit requires cleaning [85].

A major challenge in complex hydrometallurgical flowsheets with several solvent extraction circuits is *cross-contamination* of the solvent of one circuit with the solvent of another circuit [86]. This issue might become evident only after extended periods of operation, but it is extremely difficult to purify a solvent once contamination has occurred. Replacing the entire solvent inventory of a contaminated solvent extraction circuit is very expensive. The risk of cross-contamination is the reason why most complex solvent extraction flowsheets for the recovery of metals from the *black mass* of lithium-ion batteries will likely turn out to be economically unfeasible [87].

## Opportunities Offered by Cheap Renewable Energy

While issues related to the chemistry of a hydrometallurgical process are very difficult to mitigate, the situation is different when the process is considered too energy-intensive to be economically feasible. Energy-intensive processes negatively impact operating expenditures (OPEX) when energy costs are high. This was evident during the oil crisis of 1973, when soaring energy costs rendered many nickel laterite projects, based on the energy-intensive Caron or HPAL processes, uneconomical. A similar effect has been observed recently in Europe, where high gas and electricity prices have severely affected the metallurgical sector. Additionally, if the energy for a metallurgical process is derived from burning fossil fuels such as coal, heavy oil, or natural gas, the process will have a significant CO_2_ footprint. If a company is required to pay substantial CO_2_ emission taxes for its energy-intensive, fossil-fuel-based processes, this will further negatively impact the profitability of the operation.

**Fig. 6:**
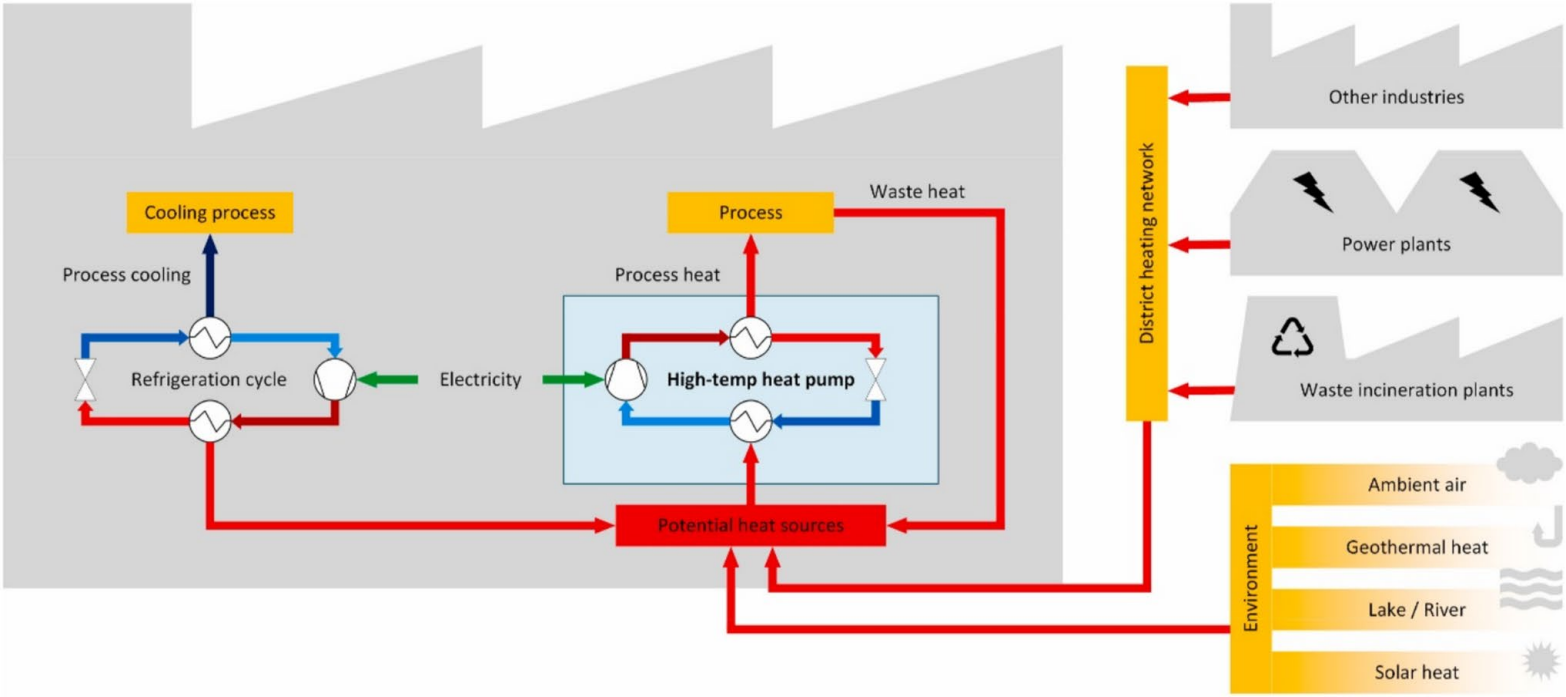
High-temperature heat pumps and potential heat sources. Reproduced from reference [91]

A major ongoing trend is the *decarbonization* of the industry, particularly through the *electrification of industrial heat demand*. By replacing fossil fuels with electricity generated from wind and solar energy, the industry can significantly reduce its greenhouse gas emissions. Many in the metallurgical industry may not realize that, theoretically, about two-thirds of industrial heat in Western countries could be supplied by electrification using currently available technologies. This figure could rise to 90% with technologies expected to reach the market within the next decade. Development is still needed for very high-temperature applications above 1000 °C, such as those required for pyrometallurgical processes and lime kilns. However, technologies for electrical heating in the temperature range relevant to hydrometallurgy are already market-ready [88]. These include heat pumps, resistance boilers, electrode boilers, and resistance heaters. Of particular interest are *high-temperature heat pumps* (HTHPs), which can deliver heat at temperatures between 90 °C and 150 °C for industrial processes (Fig. 6) [89–91]. A major challenge is the intermittency of renewable electricity, whereas most heat-demanding industrial processes are continuous. This necessitates some form of energy storage, either battery storage or thermal storage in heat batteries. Energy storage systems enable the storage of surplus energy from renewable sources, providing a stable energy supply when needed.

Renewable energy sources like wind and solar are becoming increasingly cost-effective, with their prices continuing to drop significantly over the past decade. Advances in technology, economies of scale, and increased competition have driven down the costs of producing renewable energy, making it more affordable than ever. In some instances, the abundance of renewable energy has led to situations where electricity prices drop to zero or even turn negative, meaning producers are essentially paying consumers to take the excess energy off the grid. This phenomenon occurs during periods of high renewable output and low demand, highlighting the need for improved energy storage solutions to harness and utilize this surplus effectively. Negative energy prices present a unique opportunity for energy-intensive industries to significantly reduce their operational costs. This means that industries consuming large amounts of energy, such as the metallurgical industry, can take advantage of these low or negative prices to power their operations more economically. By strategically aligning their energy consumption with these periods of surplus renewable energy, companies can significantly cut costs. Flexible energy management systems and advanced storage solutions are essential to maximize the benefits of fluctuating energy prices. For instance, in Belgium, Nyrstar is operating its tank house for zinc electrowinning as a ‘*virtual battery’* [92]. This concept allows Nyrstar to adjust its electricity usage based on the availability of renewable energy. During periods of high renewable energy production, Nyrstar can increase its zinc production, effectively storing the surplus energy in the form of a valuable product. Conversely, when energy supply is low or demand is high, they can reduce their consumption, helping to balance the grid.

Surpluses in renewable energy, leading to very low or even negative electricity prices, offer great opportunities in hydrometallurgy. This can significantly reduce the OPEX of energy-intensive hydrometallurgical processes, such as HPAL, making them more economically viable. One common challenge in hydrometallurgy is slow reaction kinetics, which can be a critical flaw. Unlike pyrometallurgical processes, hydrometallurgical processes typically operate at much lower temperatures, meaning chemical equilibrium is often not achieved, and reaction kinetics play a crucial role. Slow kinetics negatively impact the productivity of a hydrometallurgical plant, potentially preventing it from meeting its annual production targets. Reaction rates can be increased by operating at higher temperatures, which requires additional energy input and results in higher energy consumption. During periods of high energy costs, this negatively affects OPEX. However, the situation changes when electricity becomes very cheap. Slow kinetics are particularly problematic during the leaching step, as leaching is a heterogeneous chemical reaction that is inherently slower than homogeneous reactions. Leaching rates can be improved by reducing the particle size of the ore or concentrate through more intense grinding, but grinding costs increase rapidly with finer particle sizes. As grinding is the most energy-intensive unit operation in mineral processing, cheap electricity makes it more feasible to consider fine grinding as a pretreatment step. However, it is important to note that grinding costs are also influenced by the consumption of grinding media (balls or rods).

Electric resistance heating driven by inexpensive renewable energy could be used in pretreatment processes that require temperatures of several hundred degrees Celsius and were previously considered too expensive due to their higher energy intensity. These systems employ heating elements that either directly or indirectly transfer heat to a material that needs to be heated. One example of such a pretreatment process is *acid baking*. This method involves mixing the ore with a concentrated acid, typically sulfuric acid, and then heating the mixture to a temperature of usually between 300 °C and 500 °C. The heating process facilitates the conversion of metal compounds into water-soluble forms. After baking, the material is subjected to a water leaching step, where the soluble metal compounds are dissolved and separated from the insoluble residue. Acid baking is particularly effective for processing refractory minerals and complex sulfide ores, as it enhances the leachability of metals such as copper, zinc, and rare-earth elements [93, 94].

## Circular Hydrometallurgy and Lindy-Proof Processes

**Fig. 7:**
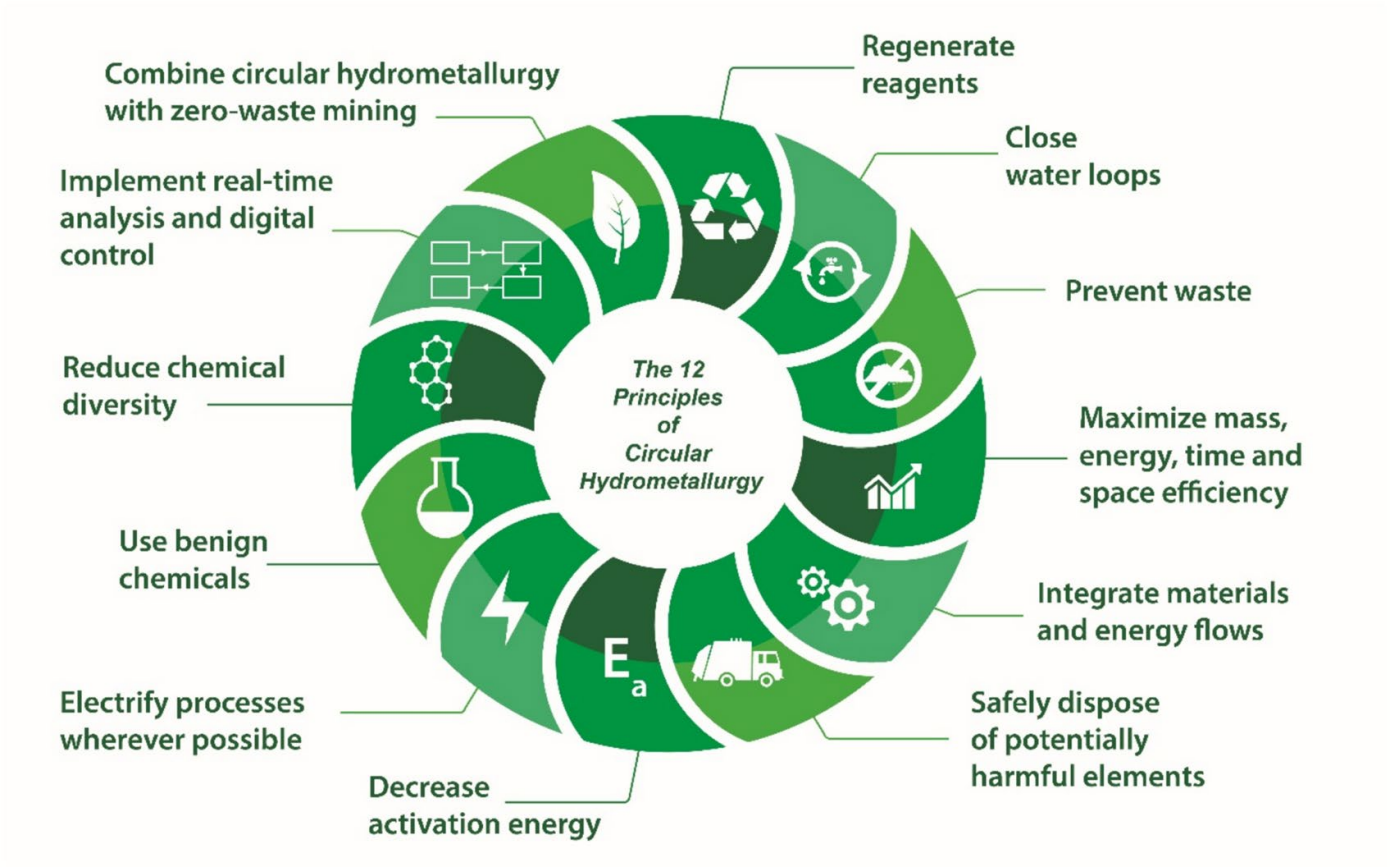
The twelve principles of circular hydrometallurgy. Reproduced from reference [7]

The framework of circular hydrometallurgy and its twelve principles was originally developed to make hydrometallurgical processes more sustainable by reducing reagent and energy consumption (Fig. 7) [7]. However, these twelve principles also improve the economical feasibility of hydrometallurgical processes and enhance the chance that a hydrometallurgical process becomes Lindy-proof. Measures that reduce operational expenditures (OPEX) will obviously affect the profitability of an operation.

Efficient and cost-effective regeneration of reagents can positively impact OPEX. Achieving inexpensive regeneration can have several beneficial effects on process economics. Increased regeneration of reagents reduces the need to purchase acids, bases, or other reagents. It also decreases the company’s dependence on external supplies, making it less vulnerable to price fluctuations and supply risks.

The principles of circular hydrometallurgy, such as closing water loops, preventing waste, safely disposing of potentially harmful elements, using a limited number of benign elements, and achieving zero-waste mining, not only enhance safety, health, and environmental outcomes but also help companies secure their *social license to operate* (SLO). This, in turn, facilitates obtaining the necessary environmental and other permits for their operations.

The principle “Electrify processes wherever possible” was initially formulated with electrometallurgical processes such as electrowinning and electrorefining of metals in mind, as well as electric-field-driven membrane processes like bipolar membrane electrodialysis (BMED) and the electrochemical regeneration of reducing and oxidizing reagents. However, this principle can be extended to the electrification of industrial heat in hydrometallurgy, as described in the previous section. High-temperature heat pumps could drive endothermic processes. For instance, solvent extraction equilibria can often be shifted by changing the temperature, allowing the extraction process to occur at room temperature and the stripping of the extracted species from the loaded organic phase at elevated temperatures (60 to 80 °C). Shifting chemical equilibria by changing the temperature can help reduce reagent consumption. Discussions about using temperature as a process variable in hydrometallurgy generally focus on heating and performing processes at elevated temperatures. However, it is also possible to perform processes at sub-ambient temperatures, such as freeze crystallization [95, 96]. Salts with a very strong temperature-dependence of their solubility in aqueous solutions can often be efficiently removed by strongly cooling the solution. For instance, the solubility of Na_2_SO_4_·10H_2_O is 19.19 g/L at 20 °C, but only 4.56 g/L at 0 °C. Wide availability of cheap electricity of renewable resources could help to promote the use of cooling rather than heating to shift chemical equilibria in hydrometallurgy.

The principles of maximizing mass, energy, time, and space efficiency (*i.e.*, process intensification), reducing activation energy, and implementing real-time analysis and digital control, can all positively impact process economics. This will lower OPEX and increase the profitability of the hydrometallurgical process. For instance, by carefully monitoring the leaching process, it is possible to avoid consuming excessive amounts of reagents, resulting in the same benefits as those mentioned for reagent regeneration: lower dependence on external supplies and reduced susceptibility to price fluctuations and supply risks. The principle of process intensification can also lead to lower CAPEX by reducing the size of reactors and other equipment in the hydrometallurgical plant, while still achieving the same nameplate capacity.

The principle of “Prevent waste” emphasizes not only avoiding waste but also maximizing the recovery of all valuable metals in mined ore. By converting these metals into marketable products, additional revenue can be generated. Harris provides a hypothetical example, illustrating how not only nickel and cobalt, but also iron, aluminum, and magnesium, could be recovered from nickel laterites [97]. Conversely, Canterford warns that if the project does not make commercial sense based on the nickel cash flow with a nominal cobalt credit then the presumed financial benefits from additional byproducts will just be an illusion [79].

## Conclusions

Because new hydrometallurgical process plants are so capital-intensive, company owners try to minimize financial risks by selecting existing commercial technologies for new plants. Therefore, the Lindy effect holds for the metallurgical industry: the older the technology, the longer it is expected to last. Well-established hydrometallurgical processes that are already more than 100 years old, such as the Bayer process for extracting alumina from bauxite, the cyanidation process for gold recovery, and the roast-leach-electrowinning process in the zinc industry, are not expected to be abandoned in the near future. This does not mean that new technologies have no chance of being implemented by the industry, but someone must be willing to take the risk to demonstrate the process on a large pilot scale, as shown by the history of the leach-solvent extraction-electrowinning process for oxidic copper ores. Otherwise, the cycle of “the new technology is not implemented because it is still unproven” cannot be broken.

Researchers in academia must realize that it is naïve to assume that metallurgical companies will be enthusiastic about new hydrometallurgical flowsheets found in peer-reviewed journals. Too often, these studies offer solutions to non-existent problems. Nevertheless, the authors of this paper are convinced that applying the principles of circular hydrometallurgy can reduce the operating costs of existing hydrometallurgical processes and make them more environmentally sustainable [7]. At the same time, these principles can inspire the development of more sustainable technologies in hydrometallurgy. Much is expected from the electrification of industrial heat in the metallurgical industry.

## References

[1] Goldman A (1964) Lindy’s Law. The New Republic, June 13, 34–35

[2] Mandelbrot BB (1983) The fractal geometry of nature. Henry Holt and Company, New York

[3] Taleb NN (2012) Antifragile: things that gain from disorder. Random House, New York

[4] Eliazar I (2017) Lindy’s Law. Physica A 486:797–805. 10.1016/j.physa.2017.05.077

[5] Urenda J, Aguilar S, Kosheleva O, Kreinovich V (2023) Fuzzy techniques, Laplace indeterminacy principle, and maximum entropy approach explain lindy effect and help avoid meaningless infinities in physics. In: Ceberio M, Kreinovich V (eds) Decision making under uncertainty and constraints: a why-book. Springer, Cham, pp 141–152

[6] Taleb NN (2018) Skin in the game: hidden asymmetries in daily life. Random House, New York

[7] Binnemans K, Jones PT (2023) The twelve principles of circular hydrometallurgy. J Sustain Metall 9:1–25. 10.1007/s40831-022-00636-3

[8] Agricola G (1556) De Re Metallica Libri XII, Translated by H.C. Hoover and L.H. Hoover, Dover Publications Inc., New York, 1912; reprinted Dover Publications Inc., New York, 1950. Froben, Basel, Switzerland

[9] Prescher H (1994) Dr. Georgius Agricola 1494–1555. GeoJournal 32:85–89. 10.1007/BF00812493

[10] Agricola G (2011) The Project Gutenberg EBook of De Re Metallica. https://www.gutenberg.org/files/38015/38015-h/38015-h.htm

[11] Brandl H (2001) Microbial leaching of metals. In: Biotechnology Set. John Wiley & Sons, Ltd, pp 191–224

[12] Faraday M (1834) Experimental researches in electricity.-Seventh Series. Philos Trans Royal Soc 124:77–122. 10.1098/rstl.1834.0008

[13] Elkington JB (1865) Manufacture of Copper (British patent 2838)

[14] Elkington JB (1869) Improvements in the manufacture of copper and in separating other metals therefrom (British Patent 3120)

[15] Elkington JB (1870) Improvement in the manufacture of copper and in separating other metals therefrom (US Patent 100131)

[16] Wraith AE, Mackey PJ, Protheroe Jones R (2019) Origins of electrorerefining: birth of the technology and the world’s first commercial electrorefinery. In: Proceedings of the 58th conference of metallurgists, Hosting Copper 2019. pp 1–15

[17] Wraith AE, Mackey PJ, Horton RW (2018) The Elkington specimen of cathode copper. Miner Process Extr Metall Rev 127:221–227. 10.1080/03719553.2017.1376141

[18] Hiskey JB (2019) The historical development of electrolyte additives and their specific role and influence on cathode quality. In: Proceedings of the 58th Annual Conference of Metallurgists, Hosting the 10th International Copper Conference 2019; Vancouver, BC, Canada. 21 August 2019. Canadian Institute of Mining, Metallurgy and Petroleum

[19] Schlesinger M, Sole K, Davenport W, Alvaer Flores GRF (2022) Extractive metallurgy of copper, 6th edn. Elsevier, Amsterdam

[20] Moebius B (1885) Process for the separation of metals (US Patent 310302)

[21] Habashi F (1999) One hundred years of cyanidation. In: Readings in historical metallurgy—Volume 1: changing technology in extractive metallurgy. pp 78–85

[22] Fivaz CE (1988) Presidential address: how the MacArthur-Forrest cyanidation process ensures South Africa’s golden future. J South Afr Inst Min Metall 88:309–318. 10.10520/AJA0038223X_1855

[23] Monhemius AJ (1996) Hydrometallurgy- the clean solution for metal production. In: Clean technologies for the mining industry, Proceedings of the 3rd International Conference. University of Concepcion, Chile, pp 113–124

[24] Hilson G, Monhemius AJ (2006) Alternatives to cyanide in the gold mining industry: what prospects for the future? J Clean Prod 14:1158–1167. 10.1016/j.jclepro.2004.09.005

[25] Aylmore MG (2016) Chapter 27 - alternative lixiviants to cyanide for leaching gold ores. In: Adams MD (ed) Gold ore processing, 2nd edn. Elsevier, pp 447–484

[26] Habashi F (2005) A short history of hydrometallurgy. Hydrometallurgy 79:15–22. 10.1016/j.hydromet.2004.01.008

[27] Habashi F (1988) 100 years of the Bayer process for alumina production. CIM Bull 81:70–76

[28] Habashi F (1995) Bayer’s process for alumina production–a historical perspective. Bull Hist Chem 17(18):15–19

[29] Tabereaux AT, Peterson RD (2014) Chapter 2.5 - Aluminum Production. In: Seetharaman S (ed) Treatise on process metallurgy, Volume 3: Industrial processes. Elsevier, Boston, pp 839–917

[30] Borra C, Mermans J, Blanpain B et al (2016) Selective recovery of rare earths from bauxite residue by combination of sulfation, roasting and leaching. Miner Eng 92:151–159. 10.1016/j.mineng.2016.03.002

[31] Evans K (2016) The history, challenges, and new developments in the management and use of bauxite residue. J Sustain Metall 2:316–331. 10.1007/s40831-016-0060-x

[32] Cooper RMG, Krysa B, Poulter SB (2010) The ideal zinc refinery flow sheet. In: PbZn 2010 : papers originally presented at Lead-Zinc 2010, held in conjunction with COM 2010 ; [held from October 3 to 6, 2010 in Vancouver, British Columbia.]. pp 283–290

[33] Kurama H (2023) Zinc extraction; in brief review from past to present. In: Kaya M (ed) Recycling technologies for secondary Zn-Pb resources. Springer International Publishing, Cham, pp 51–69

[34] Sinclair RJ (2005) The extractive metallurgy of zinc. Australasian Institute of Mining and Metallurgy, Carlton, Victoria (Australia)

[35] Simons CS (1973) The interface between the research laboratory and a profitable metals processing plant. In: Proceedings of the International Symposium on Hydrometallurgy, Chicago, Illinois, February 25–March 1, 1973 (Editors: D. J. I. Evans and R. S. Shoemaker). The American Institute of Mining, Metallurgical, and Petroleum Engineers, New York

[36] Crane RA, Sapsford DJ (2018) Towards greener lixiviants in value recovery from mine wastes: efficacy of organic acids for the dissolution of copper and arsenic from legacy mine tailings. Minerals. 10.3390/min8090383

[37] Schmitz D, Prasetyo H, Birich A et al (2024) Co-precipitation of metal oxalates from organic leach solution derived from spent lithium-ion batteries (LIBs). Metals 14:80. 10.3390/met14010080

[38] Astuti W, Hirajima T, Sasaki K, Okibe N (2016) Comparison of effectiveness of citric acid and other acids in leaching of low-grade Indonesian saprolitic ores. Miner Eng 85:1–16. 10.1016/j.mineng.2015.10.001

[39] Gargul K, Boryczko B, Bukowska A et al (2019) Leaching of lead and copper from flash smelting slag by citric acid. Arch Civ Mech Eng 19:648–656. 10.1016/j.acme.2019.02.001

[40] Jadhav U, Su C, Hocheng H (2016) Leaching of metals from large pieces of printed circuit boards using citric acid and hydrogen peroxide. Environ Sci Pollut Res 23:24384–24392. 10.1007/s11356-016-7695-9 10.1007/s11356-016-7695-9 27655620

[41] Chen X, Zhou T (2014) Hydrometallurgical process for the recovery of metal values from spent lithium-ion batteries in citric acid media. Waste Manag Res 32:1083–1093. 10.1177/0734242X14557380 25378255 10.1177/0734242X14557380

[42] Pazouki M, Panda T (1998) Recovery of citric acid – a review. Bioprocess Eng 19:435–439. 10.1007/PL00009029

[43] Ashy MA, Abou-Zeid AA (1982) Production of citric acid. Zentralbl Mikrobiol 137:395–405. 10.1016/S0232-4393(82)80018-8

[44] Reena R, Sindhu R, Athiyaman Balakumaran P et al (2022) Insight into citric acid: a versatile organic acid. Fuel 327:125181. 10.1016/j.fuel.2022.125181

[45] Wang J, Cui Z, Li Y et al (2020) Techno-economic analysis and environmental impact assessment of citric acid production through different recovery methods. J Clean Prod 249:119315. 10.1016/j.jclepro.2019.119315

[46] Binnemans K, Jones PT (2023) Methanesulfonic acid (MSA) in hydrometallurgy. J Sustain Metall 9:26–45. 10.1007/s40831-022-00641-6

[47] Binnemans K, Jones PT (2023) Ionic liquids and deep-eutectic solvents in extractive metallurgy: mismatch between academic research and industrial applicability. J Sustain Metall 9:423–438. 10.1007/s40831-023-00681-6

[48] Binnemans K, Jones PT, Blanpain B et al (2013) Recycling of rare earths: a critical review. J Clean Prod 51:1–22. 10.1016/j.jclepro.2012.12.037

[49] Dhawan N, Tanvar H (2022) A critical review of end-of-life fluorescent lamps recycling for recovery of rare earth values. Sustain Mater Technol 32:e00401. 10.1016/j.susmat.2022.e00401

[50] Kuger L, Franzreb M (2024) Design of a magnetic field-controlled chromatography process for efficient and selective fractionation of rare earth phosphors from end-of-life fluorescent lamps. ACS Sustain Chem Eng 12:2988–2999. 10.1021/acssuschemeng.3c05707

[51] Perrin MA, Dutheil P, Wörle M, Mougel V (2024) Recovery of europium from E-waste using redox active tetrathiotungstate ligands. Nat Commun 15:4577. 10.1038/s41467-024-48733-z 38830854 10.1038/s41467-024-48733-z PMC11148158

[52] Tunsu C, Petranikova M, Gergoric M et al (2015) Reclaiming rare earth elements from end-of-life products: a review of the perspectives for urban mining using hydrometallurgical unit operations. Hydrometallurgy 156:239–258. 10.1016/j.hydromet.2015.06.007

[53] Binnemans K, Jones P, Blanpain B et al (2015) Towards zero-waste valorisation of rare-earth-containing industrial process residues: a critical review. J Clean Prod 99:17–38. 10.1016/j.jclepro.2015.02.089

[54] Zhu M, Wang Y, Zheng C et al (2024) Near-zero-waste processing of jarosite waste to achieve sustainability: a state-of-the-art review. J Environ Manag 370:122396. 10.1016/j.jenvman.2024.122396 10.1016/j.jenvman.2024.122396 39244925

[55] Singh VK, Manna S, Biswas JK, Pugazhendhi A (2023) Recovery of residual metals from jarosite waste using chemical and biochemical processes to achieve sustainability: A state-of-the-art review. J Environ Manag 343:118221. 10.1016/j.jenvman.2023.118221 10.1016/j.jenvman.2023.118221 37245308

[56] Spooren J, Binnemans K, Björkmalm J et al (2020) Near-zero-waste processing of low-grade, complex primary ores and secondary raw materials in Europe: technology development trends. Resour Conserv Recycl 160:104919. 10.1016/j.resconrec.2020.104919

[57] Akcil A, Akhmadiyeva N, Abdulvaliyev R et al (2018) Overview on extraction and separation of rare earth elements from red mud: focus on scandium. Miner Process Extr Metall Rev 39:145–151. 10.1080/08827508.2017.1288116

[58] Monhemius AJ (2014) A changing environment: Reflections on 50 years of hydrometallurgy. Hydrometallurgy 2014:1–7

[59] Piret NL (2009) Will today’s needs promote copper concentrate hydroprocessing? Update and perspectives. World Metall - ERZMETALL 62:344–365

[60] Monhemius AJ (2017) The iron elephant: a brief history of hydrometallurgists’ struggles with element No 26. CIM J 8:197–206. 10.15834/cimj.2017.21

[61] Radmehr V, Koleini SMJ, Khalesi MR, Tavakoli Mohammadi MR (2013) Ammonia leaching: a new approach of copper industry in hydrometallurgical processes. J Institut Eng India: Ser D 94:95–104. 10.1007/s40033-013-0029-x

[62] Habashi F (2007) Abandoned but not forgotten: The recent history of copper hydrometallurgy. In: Cu2007 - Volume IV (Book I) - The John E. Dutrizac International Symposium on Copper Hydrometallurgy. Toronto, Canada, pp 2–19

[63] Baxter K (2016) Are we any closer to hydromet overtaking smelting for copper sulphide concentrates? In: Proceedings of ALTA 2016 Nickel-Cobalt-Copper sessions (Perth, Australia, 23–25 May 2016). pp 1–32

[64] Haavanlammi L (2007) Hydrocopper® for Treating Variable Copper Concentrates. In: The John E. Dutrizac International Symposium on Copper Hydrometallurgy. Canadian Institute of Mining, Metallurgy and Petroleum, pp 369–377

[65] Hyvärinen O, Hämäläinen M (2005) HydroCopper™—a new technology producing copper directly from concentrate. Hydrometallurgy 77:61–65. 10.1016/j.hydromet.2004.09.011

[66] Outotec Outotec to Perform Hydrocopper® Process Tests for Zangezur (Outotec Oyj Press Release, April 29, 2008). https://www.metso.com/corporate/media/news/2008/4/outotec-to-perform-hydrocopper-process-tests-for-zangezur/

[67] McKechnie R, McDonnell L, Martin S, Nikolic S (2024) Case study review of the Albion Process^TM^ as an effective alternative to pressure oxidation. In: Proceedings of the 56th Annual Canadian Mineral Processors Conference, January 23–25, 2024, Ottawa (Canada). pp 1–9

[68] Voigt P, Littleford D, Stieper G, Hourn M (2019) First commercialisation of the Albion Process^TM^ for Copper. In: Proceedings of the 58th Annual Conference of Metallurgists Hosting the 10th International Copper Conference, Paper no. 576516. pp 1–12

[69] O’Callaghan JO (2001) Minimising risk: the role of testwork in engineering design and project development. In: Proceedings of the ALTA 2001 Nickel-Cobalt Conference. pp 1–12

[70] Buchner GA, Stepputat KJ, Zimmermann AW, Schomäcker R (2019) Specifying technology readiness levels for the chemical industry. Ind Eng Chem Res 58:6957–6969. 10.1021/acs.iecr.8b05693

[71] Tinkler OS, Sole KC (2023) Copper solvent extraction on the African Copperbelt: from historic origins to world-leading status. J South Afr Inst Min Metall 123:349–356. 10.17159/2411-9717/2906/2023

[72] House JE (1989) The development of the LIX® reagents. Min Metall Explor 6:1–6. 10.1007/BF03402517

[73] Carlson ET, Simons CS (1960) Acid leaching Moa Bay’s nickel. JOM 12:206–213. 10.1007/BF03377968

[74] Whittington BI, Muir D (2000) Pressure acid leaching of nickel laterites: a review. Miner Process Extr Metall Rev 21:527–599. 10.1080/08827500008914177

[75] Southwick LM (2014) Don’t give up on the greenfield projects: The first 20 years of nickel extraction from Cuban laterites. In: Hydrometallurgy 2014, Volume II. Canadian Institute of Mining, Metallurgy and Petroleum, pp 849–869

[76] Southwick LM (2004) Two steps forward and one step back: a case of arrested development in laterite processing. In: proceedings of the international laterite nickel symposium - 2004. The minerals, metals and materials society, pp 635–655

[77] Southwick LM (2012) Red herrings on the path of technology development: capital destruction in the pursuit of a bad idea. In: towards clean metallurgical processing for profit, social and environmental stewardship, proceedings of the 51st annual conference of metallurgists of CIM (COM 2012) Niagara, ON, Canada. Canadian Institute of Mining, Metallurgy and Petroleum, pp 89–105

[78] Dobelli R (2013) The art of thinking clearly. Sceptre, London

[79] Canterford J (2011) Keeping projects on the rails. In: proceedings of the metallurgical plant design and operating strategies (MetPlant 2011) conference, 8–9 August 2011, Perth, Western Australia. The Australasian institute of mining and metallurgy, pp 12–18

[80] Canterford J (2013) Developing a ‘new and innovative’ process flow sheet – traps for all players of all ages. In: proceedings of the metallurgical plant design and operating strategies (MetPlant 2013) conference, 15–17 July 2013, Perth, Western Australia. The Australasian institute of mining and metallurgy, pp 1–5

[81] Sun Q, Yang L, Huang S et al (2018) Mechanism of poisoning hydroxyoximes by cobalt in different organic systems. Hydrometallurgy 181:64–73. 10.1016/j.hydromet.2018.08.015

[82] Cole P (2002) The introduction of solvent-extraction steps during upgrading of a cobalt refinery. Hydrometallurgy 64:69–77. 10.1016/S0304-386X(02)00013-0

[83] Rickelton WA (1992) Novel uses for thiophosphinic acids in solvent extraction. JOM 44:52–54. 10.1007/BF03223051

[84] Rickelton W, Robertson A, Hillhouse J (1991) The significance of diluent oxidation in cobalt-nickel separation. Solvent Extr Ion Exch 9:73–84. 10.1080/07366299108918043

[85] Ritcey GM (1980) Crud in solvent extraction processing—a review of causes and treatment. Hydrometallurgy 5:97–107. 10.1016/0304-386X(80)90031-6

[86] Mackenzie M, Miller G (2022) Design and operation of solvent extraction plants using two or more extractant systems. In: proceedings of the ALTA 2022 nickel-cobalt-copper conference, Perth (Australia), 22–24 May 2022. pp 261–285

[87] Harris B (2022) A critical assessment of reprocessing the cathode “Black Mass” from spent lithium-ion batteries. In: Proceedings of the ALTA 2022 nickel-cobalt-copper conference, Perth (Australia), 22–24 2022. pp 60–79

[88] Duranni J (2024) Electrification of process heat stands to slash industry’s emissions. Chemistry World September 2024:18–21

[89] Arpagaus C, Bless F, Uhlmann M et al (2018) High temperature heat pumps: market overview, state of the art, research status, refrigerants, and application potentials. Energy 152:985–1010. 10.1016/j.energy.2018.03.166

[90] de Boer R, Marina A, Zühlsdorf B, et al (2020) Strengthening industrial heat pump innovation: decarbonizing industrial heat (white paper) https://hthp-symposium.org/high-temperature-heat-pumps/white-paper-strengthening-industrial-heat-pump-innovation/

[91] Obrist MD, Kannan R, McKenna R et al (2023) High-temperature heat pumps in climate pathways for selected industry sectors in Switzerland. Energy Policy 173:113383. 10.1016/j.enpol.2022.113383

[92] Nyrstar (2024) Virtual battery. https://www.nyrstar.com/virtualbattery

[93] Demol J, Ho E, Soldenhoff K, Senanayake G (2019) The sulfuric acid bake and leach route for processing of rare earth ores and concentrates: a review. Hydrometallurgy 188:123–139. 10.1016/j.hydromet.2019.05.015

[94] Safarzadeh MS, Moats MS, Miller JD (2012) Evaluation of sulfuric acid baking and leaching of enargite concentrates. Min Metall Explor 29:97–102. 10.1007/BF03402400

[95] Heist JA (1979) Freeze Crystallization Chem Eng 86:72–82

[96] Ma Y, Svärd M, Xiao X et al (2022) Eutectic freeze crystallization for recovery of NiSO_4_ and CoSO_4_ hydrates from sulfate solutions. Sep Purif Technol 286:120308. 10.1016/j.seppur.2021.120308

[97] Harris B (2022) Toward a sustainable metals extraction technology. In: Kalin-Seidenfaden M, Wheeler WN (eds) Mine wastes and water, ecological engineering and metals extraction: sustainability and circular economy. Springer, Cham, pp 17–28. 10.1007/978-3-030-84651-0_3

